# Zirconia Dental Implant Designs and Surface Modifications: A Narrative Review

**DOI:** 10.3390/ma17174202

**Published:** 2024-08-25

**Authors:** Michał Ciszyński, Bartosz Chwaliszewski, Wojciech Simka, Marzena Dominiak, Tomasz Gedrange, Jakub Hadzik

**Affiliations:** 1 Department of Dental Surgery, Faculty of Medicine and Dentistry, Medical University of Wroclaw, 50-425 Wroclaw, Poland; michal.ciszynski@umw.edu.pl (M.C.); marzena.dominiak@umw.edu.pl (M.D.); tomasz.gedrange1@tu-dresden.de (T.G.); 2 Faculty of Chemistry, Silesian University of Technology, 44-100 Gliwice, Poland; wojciech.simka@polsl.pl; 3 Department of Orthodontics, Technische Universität Dresden, 01069 Dresden, Germany

**Keywords:** dental, implants, zirconia, surface, modifications, corrosion

## Abstract

Titanium currently has a well-established position as the gold standard for manufacturing dental implants; however, it is not free of flaws. Mentions of possible soft-tissue discoloration, corrosion, and possible allergic reactions have led to the development of zirconia dental implants. Various techniques for the surface modification of titanium have been applied to increase titanium implants’ ability to osseointegrate. Similarly, to achieve the best possible results, zirconia dental implants have also had their surface modified to promote proper healing and satisfactory long-term results. Despite zirconium oxide being a ceramic material, not simply a metal, there have been mentions of it being susceptible to corrosion too. In this article, we aim to review the literature available on zirconia implants, the available techniques for the surface modification of zirconia, and the effects of these techniques on zirconia’s biological properties. Zirconia’s biocompatibility and ability to osseointegrate appears unquestionably good. Despite some of its mechanical properties being, factually, inferior to those of titanium, the benefits seem to outweigh the drawbacks. Zirconia implants show very good success rates in clinical research. This is partially due to available methods of surface treatment, including nanotopography alterations, which allow for improved wettability, bone-to-implant contact, and osteointegration in general.

## 1. Introduction

In the past few decades, the extensive use of and extensive research on titanium implants has resulted in them being perceived as the gold standard in oral implantology. Currently, it can be agreed that their overall osseointegration rate is very high, above 95% [1].

Despite the obvious advantages, titanium and its alloys as dental implant materials have some drawbacks, which include possible discolorations of peri-implant soft tissues, leading to esthetic problems; possible hypersensitivity; and/or allergic reactions [1]. Following titanium corrosion, titanium compounds and ions have been found in the peri-implant tissues of patients who developed peri-implantitis [2]. It is argued that such compounds may contribute to osseointegration failure. Some authors even report an accumulation of titanium compounds in regional lymph nodes, lungs, and bones after the placement of titanium plasma-sprayed implants [3]. In the case of implants made not of pure titanium but of alloys like Grade 5 titanium (Ti6Al4V), corrosion could potentially result in vanadium compounds migrating to the peri-implant tissues as well as titanium. A meta-analysis by Chun-Teh Lee et al. has shown that even 30.7% implants develop peri-implant mucositis after placement [3,4].

Those drawbacks have prompted researchers to develop a new material. The first ceramic dental implants date back to as early as 1968, when alumina (Al₂O₃) implants were developed [5]. Several implant systems were developed later, in the seventies and eighties, including the Cerasand, Pfeilstift, Bonit, and Tubingen implants. However, their poor biomechanical properties often led to fractures when loaded extra-axially [6]. At the beginning of the 1990s, a new ceramic material, yttrium oxide–partially stabilized zirconia (YPSZ), was introduced to dentistry [7]. Zirconia is a crystalline oxide of zirconium [8]. Zirconia as a mineral was discovered in 1892. Stabilized zirconia was developed in 1929 [9]. It holds good mechanical, optical, and biological properties [10]. Zirconia’s fracture resistance and bending strength are significantly higher than those of Al_2_O_3_, allowing zirconia implants to withstand occlusal forces [11]. This makes zirconia the material of choice for the fabrication of ceramic dental implants.

While reviewing the available literature, one can find information concerning zirconia implants’ properties (often in comparison to conventional titanium dental implants), different methods of obtaining zirconia implants, and possible surface modifications, as well as the effects of these variables on an implant’s clinical effects. In this article, we authors aim to summarize the available information and present the current trends concerning zirconia dental implants, focusing in particular on the possible effects of surface treatment of zirconia on the performance of zirconia dental implants.

## 2. Properties of Zirconia as an Implant Material

As was mentioned in the introduction, one of the main concerns regarding titanium as a dental implant material is the possible discoloration following gingival recessions around dental implants. As authors of this review, we believe that in most cases, such esthetic problems can be avoided or solved through the careful choice of surgical techniques in each particular clinical case. For example, proper 3D implant position and soft-tissue thickness can prevent titanium from causing visible discolorations [12,13]. Moreover, anodization, a process often applied to modify titanium implants’ surface characteristics, can also be used to change an implant’s color, which can also be helpful for avoiding grayish discoloration visible in peri-implant soft tissues [14].

Nonetheless, as zirconia (ZrO_2_), introduced to dental implantology in 2005, is a tooth-colored material, it can significantly enhance the patient’s esthetic outcome, which could, in case of complications, reduce the need for additional surgical procedures. Zirconia tends not to be visible as much as titanium through thin soft tissues [15,16].

Similarly to titanium, zirconium oxide shows high biocompatibility and osteoconductivity. The bone-to-implant contact values and osseointegration rates are similar for zirconia and titanium dental implants. While titanium has been criticized for the possible corrosion and release of titanium particles and ions into the peri-implant tissues, this does not seem to be a problem in the case of zirconia implants, which show excellent resistance to corrosion and thus cause less irritation. Titanium compounds can promote local inflammatory reactions and possibly account for the lack of osseointegration [3,17].

The polymorphic zirconia structure is present in three different crystal forms: monoclinic, tetragonal, and cubic. At room temperature, zirconia acquires a monoclinic structure, which changes into tetragonal at 1170 °C and into cubic at 2370 °C. The tetragonal structure has superior fracture toughness and flexural strength thanks to the martensitic toughening mechanism [6]. The tetragonal and cubic phases are unstable at room temperature and disintegrate while cooling. It is, nonetheless, possible to stabilize zirconia in its cubic phase by adding CaO, MgO, and Y_2_O_3_ (yttria), resulting in a material called partially stabilized zirconia (PSZ). This combines all three phases. Tetragonal zirconia polycrystals (TZP) can be obtained by adding Y_2_O_3_ at room temperature. Yttria-stabilized TZP (Y-TZP) have low porosity, high density, significant bending and compression strengths, high fracture toughness, and high fatigue resistance, and their form is suitable for medical applications. On top of their favorable mechanical properties, they also promote the proliferation of osteogenic cells during osseointegration. The mechanical characteristics that are measured to assess an implant’s strength are, for example, tensile strength, bending strength, Young’s modulus, Vickers hardness, and fracture toughness. A review by Takao Hanawa states that the bending strength is 1100 MPa for Y-TZP, 400 MPa for commercially pure (Grade 2) titanium, and 950 MPa for a Ti-6Al-4V alloy. The Young’s modulus values are 210, 100 and 110 GPa, respectively. Vickers Hardness measures 1200, 150–170 and 270–320 HV, respectively. The fracture toughness of Y-TZP is 6–8 MPa/m^2^. This is significantly lower than that of Grade 2 titanium (66 MPa/m^2^) and Ti-6Al-4V alloy material (50 MPa/m^2^). Tensile strength cannot be measured for naturally brittle materials like ceramics. Bending strength is satisfactory for all of the aforementioned materials. The fracture toughness of Y-TZP is significantly lower than that of titanium. As zirconia abutments are usually screwed in with titanium retentive screws, this leads to zirconia abutments fracturing before the abutment screw. It is, however, worth noting that Y-TZP’ fracture toughness is still significantly higher than that of Al_2_O_3_ (3.1–5.5 MPa/m^2)^. Young’s modulus is important, as there is no periodontal ligament between the implant and bone. High values of Young’s modulus make it impossible for a material to absorb occlusal forces, which are then conducted directly to alveolar bone. A large Young’s modulus makes it difficult to fasten prosthetic abutment screws. Therefore, a problem with fixation can be encountered when employing Y-TZP by screwing [9,18,19].

Mechanical characteristics are important as they correlate directly to implant failures caused by possible fractures. Zirconia implants are often considered inferior to titanium ones for fear of them fracturing and requiring removal. A systematic review and meta-analysis by Bethke et al. concluded that despite some studies showing very promising results for two-piece zirconia implants, one-piece implants are in general more fracture-resistant. Moreover, implants made of alumina-toughened zirconia have been shown to be more fracture-resistant than implants made from Y-TZP [20].

Regarding osteoconductivity, it has been suggested that zirconia implants might display favorable properties compared to titanium implants. Various studies have been conducted and it has been, in fact, confirmed that zirconia as a material is osteoconductive. No clear advantage has, however, been documented over titanium surfaces [3,11].

Zirconia can however be associated with less biofilm formation than titanium, which might further decrease the risk of peri-implantitis [21]. This fact is associated with the lower surface energy and surface wettability of zirconia [22]. It has also been proven that early-formed (3-day-old) biofilms show greater accumulation on titanium rather than zirconia implants, while 14-day-old biofilms were comparable [21]. Rough or hydrophobic surfaces can, in general, increase bacterial adhesion, which suggests that different surface treatments (described in further parts of this article) of dental implants can influence biofilm formation. This appears to be true regardless of the material used. The same applies for the implant’s design—its macrostructure also affects bacterial colonization [21,23,24].

## 3. Manufacturing Zirconia Dental Implants 

The vast majority of zirconia-based dental implants are produced utilizing subtractive manufacturing methods, such as machining and milling; however, progressive processes like additive manufacturing are more promising for the future, alongside the development of digital dentistry, which gives a green light to the further progression of perfecting and polishing CAD/CAM individual zirconia implant solutions [25].

Regarding traditional subtractive manufacturing methods for zirconia implants, one can list the following techniques: dry pressing (uniaxial compaction), cold isostatic pressing, and hot isostatic pressing.

The predominant goal of these techniques is to decrease the volumetric percentage of void spaces in the block of material to the lowest possible amount during the compaction process. These empty fragments, called pores, tend to act in contradiction to the desirable physical properties of zirconia ceramics, decreasing its hardness and stiffness [26].

The first technique—uniaxial compaction—is based on hydraulic compression of the zirconia powder into a semi-finished product, which is called a blank. A lack of consistency and homogeneity leads to variable and unpredictable mechanical properties. Uniaxially pressed 3Y-TZP blanks, because of their properties, can be used successfully in manufacturing single-point prosthetic solutions, like dental crowns; however, their usage in manufacturing bridges has its limitations. The greatest asset of dry pressing is the low costs of its machinery and operation in comparison to other methods. Considering the previously mentioned characteristics, dry pressing is widely used as a preliminary stage, prior to introducing isostatic pressing as a final stage of manufacturing the product, hence eliminating the heterogeneities in the material [26].

In the cold isostatic pressing (CIP) method, a high-pressure liquid container is utilized to compress ceramic powder that is embodied inside a pliable form. CIP produces more homogenic and higher-density zirconia blanks compared to uniaxial compaction, which leads to the production of mechanically more durable blanks and enables a simplified and additionally efficient sintering process of the zirconia [27]. On the other hand, final products of the CIP method lack precision; thus, extra milling is required afterwards in order to produce more refined shapes, which causes material wastage [28].

The general idea of hot isostatic pressing (HIP) is quite akin to the concept of the cold isostatic pressing method; however, a main difference between these two processes is that HIP makes use of hot gasses (with temperatures ranging from 1300 °C to 1600 °C), like argon, compressed under high pressure, instead of the liquid utilized in CIP. Final products of the HIP method tend to be sintered, and thus they do not need any additional, complementary procedures. While costly in use, the HIP method fabricates product of the highest toughness of all three methods; that is why milling is required to achieve a desired shape afterwards. In conclusion, the hot isostatic pressing method is said to be the most costly technique when it comes to producing zirconia blanks because of its high running costs and increased cycle times. Nevertheless, different approaches have been explored by manufacturers with the aim of simplifying the whole process and refining the cycle parameters and cycle times, thus reducing the overall cost and enabling it to be more accessible for an average patient [28,29].

A constantly growing novelty in the field of manufacturing processes for zirconia oxide implants is exploiting CAD/CAM (Computer-Aided Design/Computer-Aided Manufacturing) methods. This is seen as a prospective changing factor in the near future for dental ceramics. These procedures are classified as a subtractive manufacturing technology, which allows a dental technician to digitally prepare a virtual project using a computer and then transfer this information to a milling machine for producing an implant from a blank. This technology possesses numerous advantages over a classic, analog approach. It diminishes possible occurrences of human error throughout the process, thus making it more predictable. The final product is of superior quality and precision, and also takes less time to manufacture. CAD/CAM technology, relying on digitally computed predictions, takes into consideration material shrinkage while sintering, which allows for better marginal adaptation of zirconia suprastructures. On the other hand, one of the very few disadvantages to this technology is its high cost; however, with constant technological development, it will eventually become within the reach of technicians, doctors, and patents [26,30].

Recent studies and developments in the field of CAD/CAM production processes for zirconia implants have resulted in the appearance of root-analog implant systems. They allow for the immediate implantation of an implant, mimicking the shape of an extracted tooth. An alternative way of acquiring the desired shape is the copy milling process [31].

Another intriguing branch being developed and researched involves employing additive manufacturing methods for producing zirconia oxide (3Y-TZP and ATZ) implant solutions. Currently, two technologies can be used for this procedure—direct light processing and laser-based stereolithography. New ways and opportunities open for manufacturers with these methods since they let them create implants of complex surface topography, thus improving the implant’s osteoinductive properties, while simultaneously eliminating the risk of impairing the implant’s surface through post-processes like sandblasting or acid-etching. This, being one of potential benefits of additive manufacturing, improves the firmness and endurance of zirconia oxide implants, casting a bright light for future development and research in the field of additive-manufactured ceramic solutions [32].

## 4. Surface Modifications

Many different factors affecting the results of implantation have been extensively studied and described in the scientific literature. These may include patient-specific medical conditions, medication, and biological characteristics such as the width, height, and thickness of the peri-implant-attached gingiva, bone mineral density, and vitamin D levels [33,34,35,36].

Other factors influencing osseointegration can be related directly to implant design and surface treatment. These include topography, roughness, and wettability [37].

The implant’s topography has been proven to affect the osseointegration process though the means of primary stability, sealing, and marginal bone level maintenance. These finding have led manufacturers to continuous changes over the years. Surface topography can be divided into three levels: macrotopography (on a scale ranging from 10 μm to mm), microtopography (1–10 μm), and nanotopography (1–100 nm). Macrotopography is known to affect implant stability, while microtopography can influence bone-to-implant contact, the pace of osseointegration, and the extent of adhesion between mineralized bone and the implant surface. Nanotopography is believed to affect protein adsorption and cell adhesion [37,38]. Different types of surface-modifying methods, such as UV light exposure, heat treatment, and reactive plasma treatment, can change the surface’s wettability, thus promoting the process of cell migration along the implant’s surface, and consequently shortening the osseointegration period to about a month [39].

Surface roughness has been shown to affect bone-to-implant contact, healing rates, and osteogenic responses. According to a classification created in 2009, one can distinguish smooth (machined) surfaces when the average roughness over a measurement area (Sa) is less than 0.5 μm, minimally rough surfaces when the Sa ranges from 0.5 to 1 μm, moderately rough surfaces when the Sa is 1–2 μm, and rough surfaces when the Sa is ≥2 μm [38]. Later, many studies aimed to discover the possible responses of bone and soft tissues to varying levels of implant roughness. It has been shown that fibroblasts displayed enhanced proliferation and spread on smooth surfaces [40,41]. Regarding bone cells’ behavior, it is currently agreeable that a moderately rough surface (Sa 1–2 μm) is ideal for achieving the best osteogenic effect [42,43,44,45]. Some researchers have suggested that such surfaces may promote bacterial colonization and peri-implantitis [46]. Further studies have, however, shown no evidence of an increased risk of peri-implantitis for moderately rough surfaces [45].

Surface wettability is believed to affect the adhesion of macromolecules to surfaces, the interaction of hard and soft tissues with the surfaces, biofilm formation, and clinical osseointegration rates. Surfaces can be, in general, divided into two groups according to wettability: hydrophilic and hydrophobic. Wettability is assessed by measuring the contact angle (CA) between a solid surface and a liquid when they meet. The lower the CA, the higher the wettability of a surface. In general, surfaces characterized by CA values higher and lower than 90° are considered hydrophobic and hydrophilic, respectively. The existing literature concerning wettability is relatively scarce; nonetheless, it is believed that wettability may promote tissue healing and modulate cellular adhesion levels [37,47,48].

Many processes have been tested to make titanium and zirconia implants’ surfaces promote osseointegration as much as possible. The surface modifications of titanium implants are more numerous, as they have been in use for a significantly longer time than zirconia implants. Surface treatment options only applicable to titanium include anodizing, hydroxylation, and plasma oxidation. Anodization results in a TiO_2_ layer being deposited onto the implant’s surface, which, in turn, enhances gingival fibroblast deposition, adhesion, and proliferation; improves osteoblast adhesion; and provides favorable bone-to-implant contact and success rates. Hydroxylation leads to an increase in hydrophilicity and osseointegration rates through the means of bone-to-implant contact, increased bone density, enhanced cell attachment, and osteoblast differentiation. Plasma oxidation has been shown to lead to an increase in removal torque and bone-to-implant contact. Some surface modification techniques, however, have also been applied to zirconia implants. These processes can be of a physical or chemical nature and include machining, acid-etching, sandblasting, laser modification, coating, and UV treatment [15].

Currently, a strong emphasis is put on improving and developing zirconia implant surfaces as well, especially making them additionally rough and operative. It is possible to successfully enhance cellular responses, and thus the cells’ function and reaction to the implant surface, by utilizing chemical, physical, and thermomechanical means [39]. Machine treatment of zirconia surfaces results in an average roughness around 0.96 μm. Machined zirconia implants display similar values of bone-to-implant contact to those of titanium implants, at 33.74–84.17% and 31.8–87.95%, respectively. Machined zirconia surfaces have shown a significant decrease in biofilm thickness compared to machined titanium. The osteoblast proliferation on machined titanium, however, is significantly better than that on machined zirconia. The early generation of implants used before the 1990s mostly had machined surfaces. As was mentioned earlier, a more solid bone fixation can be achieved with higher levels of surface roughness, namely above 0.96 μm. For this reason, further surface modification methods have been developed to increase the roughness, bone-to implant contact, and osseointegration rates [15,49].

**Figure 1:**
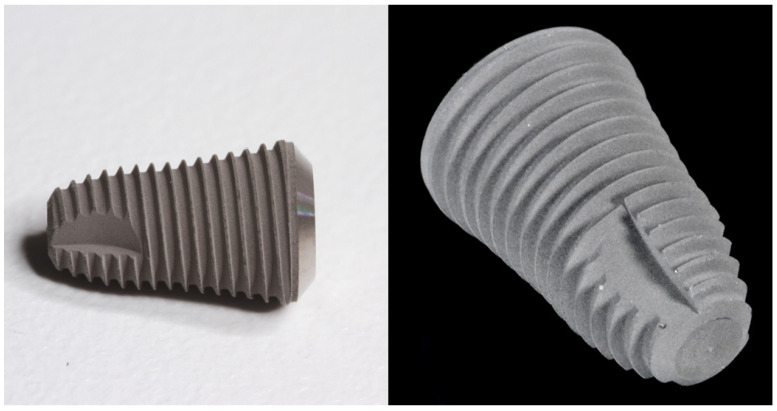
An example of an implant utilizing an SLA titanium surface [Dentium Superline 2, Dentium, Suwon, Republic of Korea].

Sandblasting and acid-etching were originally used on titanium implants to increase the surface area for osseointegration, and the resulting surface was called an SLA (sandblasted, large-grit, acid-etched) titanium surface (Figure 1). Similar processes can be used in the case of zirconia implants [50,51]. One study by Aifang Han et al. compared the characteristics of zirconia surfaces treated with grit-blasting with 110 μm silica-coated alumina particles (group GB), zirconia surfaces etched with 40% hydrofluoric acid for 25 min at 100 °C (group HF), zirconia surfaces that were grit-blasted as well as etched (group GBHP), and untreated zirconia (group C). The results showed that etching zirconia results in a desirable roughness regardless of whether it has been grit-blasted (R_a_ = 1.47 ± 0.04 μm for group HF and R_a_ = 1.49 ± 0.05 μm for group GBHF). The surface roughness in group GB measured 0.56 ± 0.05 μm, which is not satisfactory for implant purposes. Acid-etching was also shown to increase wettability. Moreover, contrary to the SLA titanium surface, acid-etching the zirconia vastly prohibited *S. sanguis* and *P. gingivalis* biofilm maturation, regardless of whether the surface had been grit-blasted or not [15,52]. In a systematic review by Gul et al., one-piece zirconia dental implants were shown to have promising 5-year clinical outcomes (95–98, 4% survival rates) regardless of their varying lengths and diameters. In the same study, acid-etching showed significantly better clinical outcomes compared to other surface designs [6]. Another study has shown that blasting zirconia with larger-sized (250 μm) particles resulted in even further enhancement of osteoblast cell adhesion in vitro [15,52,53]. It has been argued that grit-blasting Y-TZP can induce compressive stress concentration, which can lead to improvements in the zirconia’s mechanical properties. It has also been shown that grit-blasting Y-TZP causes an increase in flexural strength. Unfortunately, grit-blasting can cause deep micro-cracks, which worsen zirconia’s strength to an extent that cannot be compensated by the compression. Real clinical conditions are resembled by high-static-load and fatigue tests, which cause zirconia with micro-cracks to fail. It has been described that the changes caused by grit-blasting may increase the risk of future implant fractures. Surface treatment is only one of the many reasons that may contribute to zirconia implant fractures. Nonetheless, the fear of fractures is undoubtedly one of the reasons for zirconia still being a less popular choice than titanium, which is characterized by a 20–30-times-higher fracture toughness for Grade 4 titanium. The formation of micro-cracks can be diminished by using soft and round particles for blasting. Regarding acid-etching, high concentrations of acids and a too-long process duration can damage the zirconia’s structure and decrease its flexural strength [54,55,56].

Anodizing does play a role in modifying zirconia implant structures as well as titanium ones. In vitro studies have shown a significant increase in bone mineralization factors near anodized implants in comparison to control groups. A study conducted on rats by de la Hoz et al. showed that ZrO anodized at 60 Voltz is able to promote a significant increase in cancellous bone volume, trabecular thickness, and trabecular number, and a decrease in trabecular separation [39].

The next approach for modifying zirconia surfaces involves the use of lasers. Various types of lasers and various methods have been tested to improve zirconia surfaces for implantological needs. For example, fabricating micro-structures on zirconia with a wavelength of 1064 nm, a repetition rate of 20 kHz, an average power of 18 W, a pulse duration of 50 ns, and a fluence of 14.5 J/cm^2^ leads to an increase in the surface free energy, which leads directly to an increase in wettability [48]. In vivo animal studies have concluded that modifying zirconia implants with pulses of a hundred femtoseconds, wavelengths of 795 nm, and 10 nJ energy with a repetition rate of 80 MHz could account for bone-to-implant contact and crestal resorption rates that are comparable to those of SLA titanium implants at one and three months [57]. Another animal study has shown that using fiber laser irradiation could increase the surface roughness and lead to greater bone-to-implant contact and removal torque in comparison to machined implants. Phosphatase activity, osteocalcin expression, cell proliferation, and calcification are among the processes that benefit from the laser modification of zirconia surfaces [58]. Most of the changes are, however, associated with the creation of microgrooves and surface structures, resulting in a change in roughness [15]. It is worth noting that aside from the above-mentioned surface modification methods, lasers can also be used in treating peri-implantitis. The parameters, however, need to be chosen carefully so as not to cause overheating of the peri-implant tissues [59].

**Figure 2:**
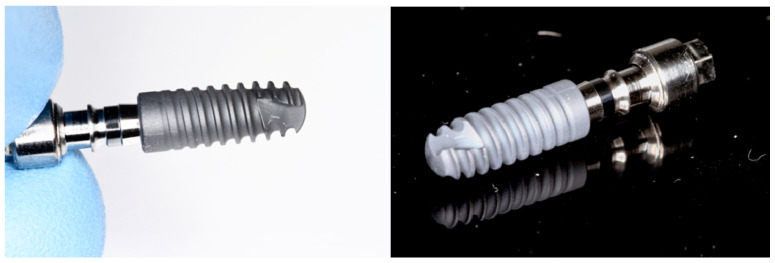
Myplant bio implant [MYPLANT GMBH, Neuss, Federal Republic of Germany] with Cerid^®^ ceramic coating.

**Figure 3:**
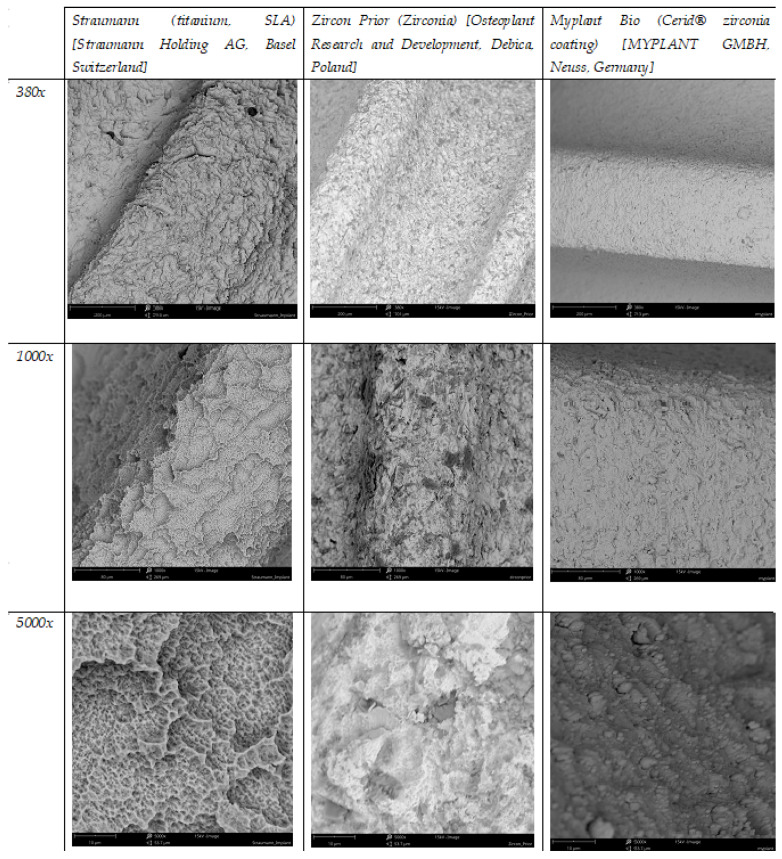
A comparison of different implant surfaces as visible through a scanning electron microscope under different magnifications.

The surface properties of zirconium oxide can also be, similarly to those of titanium, altered by applying various coatings. Regarding zirconia, these may include silica, magnesium, nitrogen, carbon, hydroxyapatite, calcium phosphate, and dopamine, all of which have been described as able to improve zirconia’s biological properties [54]. More coating types are still being developed, not only for titanium and zirconia implants but also for prosthetic abutments. Among the researched possibilities, chitosan emerges as an interesting option due to its hemostatic and antimicrobial properties, and good biocompatibility [60]. Here, it is worth noting that some commercially available implants are made of a titanium core and coated with a thin ceramic layer. The idea behind this is to determine the mechanical properties of titanium and the benefits of zirconia, while also preventing titanium’s corrosion. An example of such surface technology is the Cerid**^®^** ceramic coating on MyPlant Bio implants (Figure 2). In that particular case, the zirconium oxide coating is 4–7 μm thick. A comparison of different implant surfaces as visible under different magnifications through a scanning electron microscope can be seen in Figure 3.

Silica coatings can reduce bacterial adhesion to zirconia surfaces. Microstructured bioactive silica coatings can also assist in fibrin network formation and cell growth, and improve soft-tissue adherence. Magnesium can favor osteoblast proliferation, while the addition of nitrogen and carbon improves biological and mechanical properties of zirconia. A layer of nitrogen-doped hydrogenated amorphous carbon causes an increase in hydrophilicity and reduces bacterial adhesion. Some studies have tested coatings made of functionalized, multi-walled carbon nanotubes, which were shown to increase roughness, wettability, and cell adhesion. Hydroxyapatite, being of a similar mineral composition to bone, shows bioactive properties enhancing osseointegration, and leads to the formation of greater volumes of new bone. Calcium phosphate coating itself is too unstable to provide sufficient boding to the substrate. Because of this, studies have used tricalcium phosphate-reinforced hydroxyapatite coatings, which displayed bioresorbable and osteoconductive properties. Polydopamine as a coating has antimicrobial properties and facilitates protein adsorption and fibroblast adherence; therefore, it is very promising for improving soft-tissue integration around zirconia abutments. Graphene coatings have been shown to improve zirconia mechanical properties, leading to decreased wear and reduced occurrence of microfractures [54,61]. Nanoporous tantalum coatings can be fabricated by using tantalum nanotubes—recent studies have shown that they are able to improve osteoblasts’ differentiation and proliferation processes, thus enhancing osteointegration. Moreover, ZrO_2_/TaNS surfaces promote protein adsorption and hydrophilicity [62].

UV treatment of zirconia leads to electron excitation, which increases the surface free energy and hence also the wettability of zirconia. The available literature shows that treating zirconia with UV light for 12 or 15 min improves osteoblast attachment, proliferation, and differentiation. It also causes an increase in the osseointegration speed, bone volume, and bone-to-implant contact [15,54].

## 5. Discussion

We believe that the relatively scarce use of zirconia implants, when compared to titanium implants, can be associated with the early mentions of possible fractures. The majority of research papers focusing on zirconia implants’ survival rates have been conducted on one-piece implants. Currently, one-piece implants are hardly used, regardless of the material they are made of. One-piece zirconia implants pioneered the way for zirconia implants overall and displayed rather good clinical results, but had some significant limitations. Two-piece implants allow for the use of screw-retained restorations, which allow reintervention if necessary and are not associated with the risk of cement residues remaining in the peri-implant tissues. Moreover, they allow for the use of different prosthetic abutment designs (angulations) and more predictable reconstructive surgeries, if necessary.

There are, however, some aspects that we authors find relevant when considering early two-piece zirconia implants too. First of all, the first two-piece zirconia implants were used together with titanium prosthetic abutments. As was mentioned earlier in this text, the differences in their material properties may have led to some of the technical complications occurring—screwing titanium into a zirconia implant would cause some tensions that titanium could withstand thanks to it being more plastic and able to adapt its shape, while the same forces could cause the zirconia to fracture. Currently, zirconia implants are usually paired with zirconia abutments, so the differences appear to be less relevant. Nonetheless, the abutment is still connected using a titanium screw—more research is needed to confirm whether such a solution is suitable for long-term restorations. The position of the European Association of Osseointegration on this matter is that there is evidence of similar results for one-piece zirconia implants compared to titanium implants for the fixed replacement of one to three missing teeth. In contrast, currently available clinical data evaluating two-piece zirconia implants with an adhesively bonded implant–abutment interface suggest an inferior outcome. Data evaluating the clinical applicability of screw-retained solutions, even if revealing sufficient fracture resistance in laboratory investigations, are still missing [63].

It is worth noting here that, to omit the problems emerging as results of the high Young’s modulus of Y-TZP, a company named Patent has created a system of two-piece tissue-level zirconia implants with fiberglass abutments cemented adhesively into the implants. The abutments can be customized to any desired shape using a high-speed handpiece before a crown is fabricated and cemented onto the abutment. Such a solution aims to attenuate masticatory forces transferred from the crown to the implant and minimize the risk of implant fracture. Using a cemented restoration instead of a screw-retained one minimizes the occurrence of possible problems associated with the use of metal screws. Such implants restored with all-ceramic single crowns have demonstrated no fractures, stable soft-tissue levels, and a survival rate of 94.1% in a 9-year follow-up [64]. Studies show that this method can also be applied successfully to perform immediate implantations and immediate loading [65,66].

Regardless of all of the aspects mentioned above, recent research on two-piece zirconia implants’ clinical performance shows very good results. A meta-analysis by Roehling et al. estimated that zirconia dental implants after 5 years of observation displayed a mean survival rate of 97.2%, marginal bone loss of 1.1 mm, and a probing depth of 3 mm. This is a strong evidence that zirconia implants are a reliable treatment option [67]. Similarly to titanium, zirconia shows better clinical results when proper surface treatment techniques are applied [64,65]. A systematic review and meta-analysis by Padhye et al. concluded that there was no statistically significant difference between zirconia and titanium implant survival at 12 months, while the pink esthetic score was higher for zirconia. Zirconia implants have shown to present a lower inflammation rate compared to titanium implants due to lower bacterial attachment [68]. It is, however, worth noting that longer observations are needed to confirm zirconia implants’ good outcomes. Research papers comparing zirconia and titanium implants still appear to be limited.

The causes for peri-implantitis are similar for titanium and zirconia dental implants. The methods of treating peri-implantitis, however, differ. As zirconia has different mechanical properties, mechanical debridement cannot be applied to zirconia implants that develop peri-implantitis. Er:YAG laser therapy is the most promising option in these cases [69].

Research has shown that microbial corrosion is not more intensive for TiZr or zirconia in comparison to commercially pure titanium implants [70]. The effect of different mouthwash solutions, fluoride ions, or chlorhexidine on titanium aging and corrosion have been mentioned and it is a known fact that corroded titanium surfaces can promote bacterial colonization, reduce the ability of host cells to attach and proliferate, and impair regeneration procedures overall [71,72]. Oral bacteria are also known to play a crucial role in the corrosion of dental implants [73]. This corrosion appears important as it correlates with the occurrence of peri-implantitis. It has been shown that fluorides and toothpaste abrasives together can affect the topography and roughness of titanium, which further increases bacterial adhesion [74]. Moreover, the presence of fluoride ions damages the protective layer made of titanium oxide. This further promotes corrosion and enhances the release of titanium compounds [75]. High concentrations of fluoride ions in oral cavities lead to the formation of hydrofluoric acid (HF), which strongly promotes the corrosion of titanium [76]. Increased levels of dissolved titanium are associated with peri-implantitis [77]. Relatively low concentrations of titanium ions show cytotoxic effects on epithelial cells and cause increased monocyte migration and increased inflammation of peri-implant tissues [78,79]. Ti also stimulates osteoclastogenesis directly and indirectly through the release of inflammatory cytokines (Il-6, IL-1β, TNFα), and increases the activity of osteoclasts. The inflammatory reaction spreads into areas not in direct contact with the titanium [80,81]. Despite signs that zirconia implants can also corrode and that zirconium was in fact found in oral mucosa in gingival tissues, the literature discussing zirconia implants’ corrosion is relatively scarce [82]. Similarly, the literature concerning different factors that could potentially affect zirconia’s corrosion is almost non-existent. Some studies do, in fact, describe the mechanisms of zirconia’s wear: stress corrosion and chemical degradation. Stress corrosion is the slow transformation of surface particles to the monoclinic phase over time, while chemical degradation is a chemisorption of OH^−^ from water at the surface of zirconia grains to form Y(OH)_3_. As was written in the aforementioned study by Thomas et al., the oral cavity is an aqueous, electrochemical environment susceptible to various changes in pH, which can promote the corrosion of zirconia [83].

Oral hygiene products can contain various chemicals, whose effects on the corrosion of zirconia are not yet known. Taking this and all of the above into consideration, we authors of this narrative review believe that further research needs to be conducted on zirconia corrosion, especially in response to different concentrations of substances applied in daily oral hygiene.

## 6. Conclusions

Zirconia implants in general seem to present satisfactory mechanical properties, undoubtedly sufficient for clinical use. While some of their properties may cause difficulties, available solutions allow clinicians to provide successful treatment. Their osseointegration ability and biocompatibility are equally good or even superior to those of titanium implants. Many research papers suggest that superior esthetic outcomes, especially in the anterior region, can be achieved by using zirconia implants rather than titanium. Nonetheless, the available observations on zirconia implants are relatively scarce and more research needs to be conducted.

## Data Availability

Data can be made available on request.
